# Nonthermal Plasma Effects on Fungi: Applications, Fungal Responses, and Future Perspectives

**DOI:** 10.3390/ijms231911592

**Published:** 2022-09-30

**Authors:** Lucia Hoppanová, Svetlana Kryštofová

**Affiliations:** 1 Department of Biophysics and Electrophysiology, Institute of Molecular Physiology and Genetics, Centre of Biosciences, Slovak Academy of Sciences, Dúbravská Cesta 9, 841 04 Bratislava, Slovakia; 2 Institute of Biochemistry and Microbiology, Faculty of Chemical and Food Technology, Slovak University of Technology, Radlinského 9, 812 37 Bratislava, Slovakia

**Keywords:** antifungal effect, decontamination, fungi, nonthermal plasma, oxidative stress, spores

## Abstract

The kingdom of Fungi is rich in species that live in various environments and exhibit different lifestyles. Many are beneficial and indispensable for the environment and industries, but some can threaten plants, animals, and humans as pathogens. Various strategies have been applied to eliminate fungal pathogens by relying on chemical and nonchemical antifungal agents and tools. Nonthermal plasma (NTP) is a potential tool to inactivate pathogenic and food-contaminating fungi and genetically improve fungal strains used in industry as enzyme and metabolite producers. The NTP mode of action is due to many highly reactive species and their interactions with biological molecules. The interaction of the NTP with living cells is believed to be synergistic yet not well understood. This review aims to summarize the current NTP designs, applications, and challenges that involve fungi, as well as provide brief descriptions of underlying mechanisms employed by fungi in interactions with the NTP components

## 1. Introduction

The motivation for understanding the effect of nonthermal plasma (NTP) treatment on fungi or other microorganisms stems from the unique and complex nature of plasma and the complexity of processes triggered in the fungal cells upon interaction with the plasma components. NTP in medicine, agriculture, and food processing is used to devitalize and decontaminate various surfaces and liquids. The application of NTP could expand to biotechnology for fungal breeding and antifungal resistance management. Recently, several excellent reviews summarized achievements in the utilization of various types of NTP devices in antifungal treatment [1,2], but very few elaborated in depth on molecular mechanisms triggered by NTP [3]. At the moment, we face a lack of a better understanding of molecular mechanisms and experience some difficulties regarding the methodology influenced by many variables in the experimental setup of the plasma devices, as well as biological differences in fungal species and cell types and biological sample handling. Since plasma has gained significant attention in antifungal treatment in recent years, this review aims to equip readers with the most recent information on NTP compositions and designs, direct and indirect applications, and molecular mechanisms employed by fungi in response to NTP.

The review is organized into several chapters. Section 2 introduces the NTP systems used in the fungal treatments, plasma generation, composition, and biological mechanisms that can be triggered by plasma in fungal cells. It also summarizes biologically active reactive species present in plasma and their effects on fungi. Section 3 provides an overview of plasma applications in medicine, agriculture, food preservation, biotechnology, and the protection of cultural objects.

## 2. NTP Devices, Plasma Composition, Biologically Active Agents, and Fungal Responses

### 2.1. Plasma Classification and Configurations of NTP Systems

Plasma, considered to be the fourth state of matter, is a fully or partially ionized gas formed of charged particles (free electrons, positively and negatively charged ions), free radicals, neutral gas particles (excited atoms and molecules), photons (in the visible and UV regions of the spectrum), and the electromagnetic field [4,5,6].

**Figure 1:**
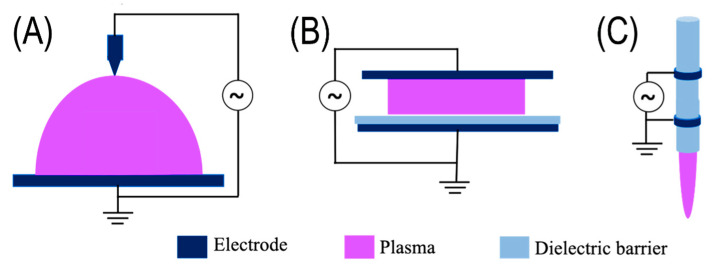
Configuration of basic NTP systems: (**A**) corona discharge; (**B**) dielectric barrier discharge; (**C**) plasma jet (adapted and modified from [14]).

Plasma is a quasi-neutral system of free electrons and ions that exhibit collective behavior. This behavior is reflected in the plasma response to deviations from neutrality and applied external electromagnetic fields and in its ability to sustain many different waveforms and oscillations. Plasma is produced by an ionization process that generates a certain number of free electrons and positive ions. If quasi-neutrality applies, the number density of electrons n_e_ is approximately equal to the ion density of n_i_, n_e_ ≈ n_i_, and n_e_ is called the plasma density. Typical plasma density units are cm^−3^. The plasma density at atmospheric pressure can vary from 10^9^ to 10^19^ cm^−3^, corresponding to a degree of ionization from 10^−10^ to 1 [7,8]. Depending on the method of formation, plasma can be high-temperature (thermal) and low-temperature (nonthermal) plasma (NTP). Thermal plasma is created by heating a gas to a temperature at which electrons are torn from atoms and ions are formed. It can reach a temperature of up to 10^6^ K. NTP is generated by an electric discharge when the generated ions reach a temperature close to the environment (maximum 340 K), which predestines NTP for use in many applications [9,10,11,12]. NTP is often referred to as nonequilibrium plasma because it is not in thermodynamic equilibrium. Nonequilibrium plasma is characterized by the temperature of electrons ranging from a few eV to 10 eV, while the temperature of heavy particles varies from room temperature to a level comparable to the electron temperature but usually lower [13]. NTP is easily formed in the air at atmospheric pressure using various discharges. In addition to air, plasma can also be created in other gases such as nitrogen, oxygen, argon, or carbon dioxide. The most commonly used electric discharges are corona discharge, dielectric barrier discharge, and plasma jet (Figure 1).

#### 2.1.1. Corona Discharge

A corona discharge (Figure 1A) can be observed as a luminous glow. Near to sharp electrodes such as thin wires, spikes, or edges in a highly non-uniform electric field with high intensities, the active region of corona and plasma generation occurs [15]. Point-to-plate geometry, which is a sharply curved electrode arranged as a counterpart to a flat one, is a typical electrode geometry. Corona discharges can be operated in direct current or pulsed mode, where the pointed electrode has a negative or positive potential [9]. Corona discharges are used in various industrial applications [16,17,18,19,20,21].

#### 2.1.2. Dielectric Barrier Discharge

Due to its configuration and flexibility of electrode shapes, dielectric barrier discharge (DBD) (Figure 1b) is one of the most commonly used plasma systems. DBD plasma is generated by a high voltage applied between two metal electrodes, which are covered with a dielectric material (glass, ceramic, or polymer), and micro-discharges do not occur [15,22,23]. These sources operate at frequencies of 50 Hz to 500 kHz, while the voltage amplitude can be up to tens of kV. The gap between the electrodes can be several µm to several cm. Volume and surface DBDs are the most well-known configurations of this arrangement used to treat biological objects. Volumetric DBD is also known as industrial corona [24]. It consists of two parallel plates in a plane, or the electrodes can be curved in the shape of a cylinder. The surface DBD is composed of parallel electrodes separated by a dielectric barrier layer, while the plasma is formed in an uneven electric field. In the surface DBD configuration, the gap between the discharges is flexible, allowing the treatment of objects of different sizes. The disadvantage of this arrangement is the device’s lifetime, which is limited by contact of plasma with the electrodes [25]. The advantages of volume and surface DBD are combined in a coplanar configuration where a dielectric barrier layer covers pairs of linear parallel electrodes with opposite polarity. Electrodes can have an area of up to a few cm^2^, which makes this type of plasma particularly suitable for processing large surfaces.

#### 2.1.3. Plasma Jet

A plasma jet is not considered a plasma discharge. It is a specific configuration of other discharges, e.g., corona discharge, DBD, and microwave discharge [15]. An auxiliary gas (usually noble gases) flows through the two electrodes generating the plasma, which pushes the plasma out of the electrodes. A stream of active particles burning as a small jet is created. A plasma jet makes a stable, homogeneous, and uniform discharge at atmospheric pressure. It is used in plasma sources called jets, torches, or pens [15]. The disadvantage is that the plasma jet is only suitable for treating small surfaces. When treating large areas, it is necessary to use several jets in a row [26].

### 2.2. Biologically Active Agents Generated by Plasma

In NTP, depending on the parameters (gas composition, humidity, and temperature), biologically active agents (BAAs) are formed as a result of many physical and chemical processes. Among BAAs generated by plasma, we include, for example, ROS (reactive oxygen species), RNS (reactive nitrogen species), UV radiation, radiation in the visible and infrared spectrum, charged particles, alternating electric field, and heat [4,9,27]. In recent years, many experiments have been conducted dealing with the importance of individual BAAs generated by plasma in the inactivation process of microorganisms [27,28,29,30]. It is difficult to objectively evaluate which plasma component is the most effective because different types of plasma sources do not have to generate BAA in the same amount, and it is always necessary to identify them. Each of these factors inactivates microorganisms independently, but they are much more effective if their synergistic effect is used [31,32,33], making NTP unique. Of all BAAs generated by NTP, ROS and RNS (RONS) are the most critical inactivating agents of plasma, and NTP has been shown to induce oxidative stress, which can result in cell damage or death [27,29,31,32,34,35,36]. RONS are responsible for several biological reactions, from intracellular DNA breaks to protein damage to outer membrane oxidation [28].

Depending on the type of plasma source used and the conditions of plasma generation, the electric field can contribute to the inactivation of microorganisms. Processes similar to electroporation and disruption of cell morphology may occur during NTP biomass treatment. Plasma treatment can break the cell membrane, which then loses integrity, resulting in the leakage of cytoplasmic components out of the cell [27,37,38].

UV radiation has mutagenic to lethal effects and is widely used in sterilizing rooms and spaces. Nevertheless, UV photons originating from the plasma play only a minor role in the inactivation process [27,29,30]. Plasma-generated UV radiation does not have such a striking impact on cells as the use of UV lamps. In addition, many microorganisms contain protective pigments, such as melanin, in the cell wall of fungi, which to some extent, can protect against UV damage [36].

The effect of NTPs and, thus, BAAs originating from plasma on biological material is dose-dependent, although “dose” is still not a precisely defined term [32]. So far, it has been found that the plasma effect on the treated biological material is more substantial with higher plasma power, more prolonged exposure of the biological material, and closer placement of the material to the plasma or the electrode surface. For example, low doses of NTP cause mammalian cells to proliferate, higher doses cause apoptosis, and even higher doses may cause necrosis [32].

### 2.3. Fungal Molecular Mechanisms in Response to NTP

NTP generated in ambient air can produce reactive species (RONS), such as free electrons, atomic oxygen, singlet oxygen (^1^O_2_), **^.^**O_2_^−^, **^.^**H, **^.^**OH, **^.^**NO, **^.^**NO_2_, O_3_, and atomic oxygen. In aqueous liquids, primary RONS react with each other, and compounds such as H_2_O_2_, NO_2_, and NO_3_ are formed. Their formation leads to extracellular and intracellular liquid acidification [39,40,41,42]. Most fungal pathogens grow well in an acidic environment but struggle in alkaline conditions [43,44]. It is well known that fungi generally have a wide pH optimum (4–9 pH units). Nevertheless, the drop in intracellular pH could contribute to maintaining membrane potential in the plasma oxidized fungal cell membrane [42].

Reactive species are believed to be a major factor responsible for the effects of plasma on living cells. Although more studies regarding the molecular action of the NTP have been published on bacteria, mammalian, and plant cells than on fungi, many of the mechanisms may be shared by different species [45]. The function of ROS has been well studied in fungal cell signaling. ROS are intracellularly produced as metabolic byproducts under normal physiological conditions during development or stress responses [46]. ROS can react in excess with biomolecules, such as proteins, lipids, and DNA, which can harm cells. Therefore, cells possess several ROS-scavenging systems. The antioxidant systems are composed of nonenzymatic and enzymatic types [47]. The major nonenzymatic antioxidant is tripeptide glutathione, which forms a disulfide bond between cysteines of two glutathione molecules, resulting in the generation of an oxidized form of glutathione. In *A. flavus*, plasma treatment led to a significant decrease in the reduced form of glutathione, indicating a potent oxidative attack during plasma treatment which likely caused depletion of the reduced glutathione [48]. In addition to glutathione, some other organic compounds in fungi exhibit scavenging properties, such as ascorbic acid, carotenoids, flavonoids, alkaloids, mannitol, and trehalose [49,50]. In addition to non-protein ROS scavengers, thioredoxin proteins, their respective reductases, and antioxidant enzymes such as catalases, superoxide dismutases, and peroxidases are involved in cellular protection against ROS. The role of the antioxidant enzymes in fungal defense in response to plasma treatment was confirmed in *A. flavus* and *S. cerevisiae* [48,51].

ROS generated by plasma sources are characterized by a short lifetime and their ability to interact with reduced functional groups of organic compounds in cells [52]. ROS oxidation of cysteine residues in proteins leads to the generation of cysteine sulfenic acid (–SOH) and disulfide bonds between two cysteines. The formation of disulfide bonds is a reversible modification. In yeast *S. cerevisiae*, transcription factor Yap1 responds to plasma treatment by rapid translocation from the cytoplasm to nucleus. The translocation is initiated by forming disulfide bonds in the protein region governing the transport into the nucleus [53]. Yap1 activates the expression of antioxidant stress response genes.

Sulfenic acid can be oxidized to sulfinic (–SO_2_H) or sulfonic (–SO_3_H) acid. This cysteine modification is, however, irreversible [54] and damaging to cells. In addition to cysteine, methionine possesses a sulfur-containing side chain susceptible to oxidation. The oxidized methionine, methionine sulfoxide, is one of the important post-translational modifications [55] that ROS can affect. At the moment, there is very little information on cysteine and methionine oxidations in fungi following plasma treatment.

The major targets of ROS from plasma are fungal cell walls and cytoplasmic membranes. FTIR analysis and electron microscopy in *Aspergillus* sp. indicated chemical (polysaccharide oxidation) and physical changes (dehydration, ruptures) in cell surface structures [42,48,56,57,58]. Currently, we do not have many studies regarding the nature of ROS interaction with cell surfaces of fungal cells and the depth they can penetrate. ROS are divided into long- and short-lived species. It was reported that the interplay of those species and their concentration gradients and penetrability with the cell surface might initiate a sequence of cell responses [42,59]. Although fungi do not synthesize polyunsaturated fatty acids, malondialdehyde (MDA) formation was determined in fungi after plasma treatment [42,48], indicating lipid peroxidation. Protein and potassium leakage and membrane potential reduction suggested the loss of membrane integrity. Damage to cell membranes inflicted by reactive species also led to mitochondrial malfunction, endoplasmic reticulum stress, defects in protein folding, and intracellular calcium increase [42,53,60,61,62].

In addition to proteins, lipids, and polysaccharides, ROS target nucleic acids. In eukaryotic cells, single-strand and double-strand break formations were reported, along with forming oxidized bases such as 8-oxodeoxyguanosine [63,64]. These breaks are subjected to DNA repair mechanisms which could result in mutations or cell death if the damage overwhelms the DNA repair machinery. Apoptosis-like markers such as chromatin condensation, phosphatidylserine presence on the outer plasma membrane, decrease in mitochondrial transmembrane potential, and cell-cycle arrest [61,65] were determined in yeasts. However, yeast mutants lacking genes for the proapoptotic proteins Yca1p, Aif1p, and Nuc1p (metacaspase, apoptosis-inducing factor, endonuclease G) did not differ significantly in sensitivity from the wildtype when treated with NTP [51]. These results indicate that fungi might have a plasma-specific type of death that does not require the activation of the fungal programmed cell death pathway.

## 3. NTP Technology in the Management of Fungal Contamination, Disease Control, Protection of Heritage Objects, and Strain Improvement

Microbial inactivation using NTP is especially suitable when traditional decontamination methods are ineffective. Since the differences in the structure and size of cells, their metabolic activity, and the ability to cope with reactive molecules in different microorganisms are not sufficiently studied, a complete generalization of the effects of plasma is not possible. Many studies confirmed the applicability of NTP for the inactivation of fungal cells (Table 1), which show lower sensitivity to NTP than bacteria [66,67,68].

Fungal cells were effectively inactivated by plasma after only a few minutes of exposure to NTP. The action mechanism is based on damage to the structure of cell envelopes and oxidation of macromolecules, similar to bacteria [81]. The level of oxidative stress induced by NTP is a critical factor for cell fate determination. Plasma-generated ROS contribute most to fungal inactivation. The NTP can induce two modes of cell death (apoptosis or necrosis) in fungal cells dependent on treatment time [71]. The most studied fungal genera include *Aspergillus* sp., *Penicillium* sp., *Fusarium* sp., and others. Šimončicová et al. [48] investigated the effect of plasma on *A. flavus* hyphae, reporting massive structural changes, increased membrane permeability, and DNA degradation. The DNA damage by plasma-induced intracellular RONS was also confirmed in *Cordyceps pruinosa* spores [77]. Julák et al. [78] observed a delay in the growth of *Aspergillus oryzae* and *Alternaria* sp. after exposure of conidia to plasma. This phenomenon is probably related to the mechanism of plasma effects on fungal cells. After nonlethal damage, revitalization processes begin restoring damaged components and functions. Yeasts, especially *Candida* sp. and *Saccharomyces* sp., are frequently used as model organisms. Tyczkowska-Sieroń et al. [73] studied changes in the genome of *Candida albicans* after exposure to a sublethal dose of plasma. They identified six single-nucleotide variants, six insertions, and five deletions and also demonstrated that, of the 19 hydrolytic enzymes, nine were inactive, nine temporarily decreased the activity, and one constitutively increased the activity after plasma exposure. Carbon assimilation and drug sensitivity were not affected by plasma. Hence, they concluded that the changes in surviving *C. albicans* cells did not impose significant danger to the environment, especially regarding drug resistance and pathogenicity.

Some microorganisms can form mono- or polymicrobial aggregates referred to as biofilms. This structure protects pathogenic microorganisms from antimicrobial agents and the immune system. According to some estimates, a pathogen biofilm is present in the body in up to 80% of diseases. *C. albicans* is one of the most common human opportunistic yeasts. Infections caused by *C. albicans* are associated with their ability to form a biofilm. Several studies proved the positive effect of plasma on biofilm inactivation [82,83,84]. The complete killing of *C. albicans* cells in the biofilm was observed after 8 min of plasma treatment [84]. A study of *A. flavus* biofilm showed that plasma treatment has detrimental effects on the biofilm structure. At the same time, it pointed out that the fungicidal effect of plasma may depend on the initial concentration of the inoculum [82].

### 3.1. Plasma Medicine

NTP generated at atmospheric pressure shows promising biomedical applications leading to the emergence of plasma medicine that includes the inactivation of bacteria, fungi, viruses, and endospores, blood clotting, wound healing, and tooth whitening. Applications in antitumor therapy are also being studied, where plasma exhibits an antitumor effect on a wide range of cancer cell lines [33,85,86,87,88].

Fungal infections cause a complex set of disease states that cause tissue destruction or may result from inflammation caused by the presence of the fungus [89]. Among the relatively common fungal diseases are candidiasis, onychomycosis, and dermatophytosis. Older people, people with organ transplants, HIV-positive people, and diabetics are especially prone to developing candidal infections [90,91]. Borges et al. [92] tested plasma jet as a possible effective tool for preventing oral candidiasis in vivo. After only 5 min, they observed a significant decrease in the viability of the *C. albicans* biofilm. Histological analyses revealed a significantly lower incidence of inflammatory changes and a substantial reduction in candidal tissue invasion in the plasma-treated group. Park et al. [93] found that 1–5 min application of no-ozone cold plasma inhibited the growth of *C. albicans* by approximately 2 log.

Dermatophytosis is a term used to describe fungal infections caused by fungi that colonize the surface of the skin, hair, or nails. The most common are representatives of the genera *Epidermophyton*, *Microsporum*, and *Trichophyton* [94]. The effect of plasma in preventing dermatophytosis was monitored with silver nanoparticles. Such treatment decreased the minimum inhibitory concentration of nanoparticles, increased mycelial permeability to nanoparticles, and increased the effectiveness of healing and suppression of disease symptoms on the skin [95]. In guinea pigs infected with *Trichophyton mentagrophytes*, plasma treatment shortened and attenuated the infection and significantly reduced the viability of the pathogen without adverse effects on the animal model [96].

Onychomycosis is a nail fungal infection that afflicts almost 6% of the population worldwide [97,98]. About 70% of these infections are caused by dermatophytes (*Trichophyton rubrum* (more than 50%), *Trichophyton mentagrophytes*, *Epidermophytonon floccosum*, *Microsporum* spp., *Trichophyton violaceum*, *Trichophyton verrucosum*, *Trichophyton krajdenii*, and *Arthroderma* spp.), about 20% are caused by non-dermatophyte molds (*Scopulariopsis brevicaulis*, *Aspergillus* spp., *Acremonium*, *Fusarium* spp., *Alternaria alternata*, and *Neoscytalidium*), and 10–20% are caused by yeasts (*Candida* spp.) [99]. Bulson et al. [100] observed the complete killing of *C. albicans* and *T. mentagrophytes* by plasma in suspension and on nails. A similar plasma effect was also observed in the inactivation of *T. rubrum*, *Trichophyton interdigital,* and *Trichophyton benhamiae* [101].

### 3.2. Plasma Food Technology and Agriculture

To fulfill the needs of an ever-growing population, it is necessary to ensure a sufficient amount of high-quality raw materials. In this case, NTP is a suitable alternative to the already used technologies [102,103,104]. Plasma has been effectively used to decontaminate various food surfaces such as fruit, vegetables, and meat (Table 2). Park et al. [105] investigated the effect of plasma on the reduction of *Cladosporium cladosporioides* and *Penicillium citrinum* on the surface of dried filefish fillets. After 20 min of treatment, they determined a 0.9–1 log reduction of CFU/g, but at the same time observed a decrease in overall sensory acceptance. Plasma treatment caused a reduction of viable fungal spores on beef jerky but harmed off-color, flavor, and overall acceptability [106]. Royintarat et al. [107] used the synergistic effect of ultrasound and plasma-activated water (indirect plasma action) to reduce microbial contamination of chicken meat. Sudheesh and Sunooj [108] used plasma to treat fresh-cut fruits and vegetables. In addition to the inactivation of the microbial cell, they observed a decrease in enzymatic activity (pectin methylesterase and polyphenol oxidase), which is also related to the browning speed. There was also a decrease in antioxidant content and antioxidant activity. Thanks to the possibility of plasma generation in liquids [109], this method is also appropriate for decontaminating water, milk, and fruit juices [110,111]. Xiang et al. [112] used plasma to inactivate the yeast *Zygosaccharomyces rouxii* in apple juice. Treatment of juice with plasma for 140 s reduced *Z. rouxii* by approximately 5 log. At the same time, plasma caused significant changes in apple juice’s pH, acidity, and color parameters, but had no effect on the content of total soluble solids, reducing sugars, and total phenols. The changes in apple juice acidity may be related to the production of acidogenic molecules such as NOx or H^+^ dissociated from H_2_O and other components in apple juice during DBD plasma treatment. No significant changes in physicochemical properties were observed in tomato juice [113]. However, several studies have demonstrated the effects of plasma on components determining food quality, such as pH, proteins and enzymes, sugars, lipids, vitamins, and others [110,114].

The safety of crops and food is critical because of the health risk and the enormous economic losses. NTP can also be used for disinfection post-harvest fruits and vegetables. DSBD plasma was effectively used to inhibit the growth of natural microbiota and the natural decay of blueberries. After less than 15 min, only modest effects of plasma on blueberry quality were observed. However, 20 min treatment resulted in severe oxidative damage to the peels [115]. Plasma treatment did not significantly change the taste, aroma, color, and texture of kumquat [116] or the color and hardness of paprika during storage [117]. The treatment of mung bean sprouts with PAW did not cause significant changes in mung bean’s total phenolic and flavonoid content and sensory properties [118]. Using a microwave plasma jet significantly increased mandarin peel’s entire phenolic content and antioxidant activity [119]. Liu et al. [120] developed a high-field plasma system at atmospheric pressure to control and keep the storage area clean and to keep plants such as vegetables, fruits, and flowers fresh for longer. The study showed that, with the help of the plasma system, fresh fruits (bananas, grapefruits) are preserved much longer compared to the conventional methods. The amount of ethane emitted during storage was also reduced. Ambrico et al. [121] found that pretreatment of cherries with plasma leads to increased resistance to subsequent fungal infection. It is also worth mentioning a study showing that NTP can degrade pesticide residues in fruits and vegetables [122].

Contamination of food with mycotoxins is a global problem. Despite implementing various measures in agriculture, the contamination of raw materials during storage and processing cannot be completely prevented. Another problem is that, due to the high stability of mycotoxins against thermal, physical, and chemical influences, it is impossible to remove them altogether during food processing [148]. Mycotoxins spoil food and feed, threaten human and animal health, and hinder international trade [149]. Approximately 25% of the world’s crops are contaminated with mycotoxins each year, resulting in enormous agricultural and industrial losses estimated in the billions of dollars. The main mycotoxin-producing fungal genera include *Aspergillus*, *Fusarium,* and *Penicillium*. While species of the genera *Aspergillus* and *Penicillium* contaminate food and feed during storage, species of the genus *Fusarium* colonize crops directly in fields and plantations [148]. NTP was effectively applied for inactivating mycotoxin producers. Therefore, questions arose about whether plasma could be used for mycotoxin degradation. Aflatoxin B1 was completely degraded after plasma treatment of corn kernels [134], approximately 73% degradation was observed on hazelnuts [137], and a 45–56% reduction was achieved on rice and wheat [136]. Hojnik et al. [150] investigated the possible cytotoxic and genotoxic potential of aflatoxin B1 (AFB1) plasma degradation products on human hepatocellular carcinoma cells. Cytotoxic and genotoxic effects of NTP-treated AFB1 compared to NTP-untreated AFB1 were not confirmed. Hoppanová et al. [57,58] investigated changes in aflatoxin and ochratoxin production in response to plasma-induced oxidative stress. Their results clearly showed that NTP can significantly reduce viable cells. However, the cells that survived the plasma treatment were able to produce mycotoxins at an increased rate in the early stages of growth and their production slowed down in the later stages of growth. From a practical point of view, this means that, even after decontamination of food with plasma, it is still necessary to follow the principles of proper and safe food storage.

Many studies point to the positive results of using plasma in agriculture (Table 2). In addition to seed disinfection, plasma can improve the germination rate of many seeds, which can lead to enhanced production [67,142,147,151,152,153,154]. It has been shown that irrigation using plasma-activated water leads to better growth of radishes, tomatoes, and peppers [155]. Changes in the seed’s surface properties were also observed, thanks to which their wettability and water absorption increased [66,67,156,157]. It was observed that just 10 s of plasma treatment changes the surface of cereal seeds from hydrophobic to hydrophilic. Due to the better wettability of the seeds, it is necessary to apply a lower volume of chemical fungicides. By combining physical (NTP) and chemical (fungicide) treatment of cereal seeds, it is possible to effectively reduce the required amount of chemical fungicide and stimulate the germination and early growth parameters of the seed [158]. NTP could be an alternative for reducing the amount of chemical fungicides used in agriculture and for the degradation of toxic chemical compounds such as phenols and azo-dyes [159].

### 3.3. Plasma and Cultural Heritage Objects

Due to their high enzymatic activity and ability to grow even at low a_w_ values, fungi can grow on paper, parchment, paintings, textiles, and other materials. Thus, they play a crucial role in damaging cultural heritage. Among the most widespread fungal genera damaging historical objects are *Alternaria* sp., *Aspergillus* sp., *Aureobasidium pullulans*, *Fusarium* sp., *Mucor* sp., *Penicillium* sp., *Botrytis cinerea*, *Trichoderma harzianum* a *Trichoderma viride*, *Cladosporium cladosporioides*, and *Epicoccum nigrum* [160]. NTP is a possible and effective method of inactivating fungal contamination to effectively save historical artifacts. DBD plasma is used to stabilize documents containing iron gall inks [161]. Low-temperature ADRE (atmospheric discharge with runaway electron) plasma can decontaminate the surfaces of various lignocellulosic materials from five types of filamentous fungi (*A. alternata*, *Cladosporium herbarum*, *Penicillium chrysogenum*, *A. niger*, and *Trichoderma atroviride*). The least sensitive to ADRE plasma treatment were the filamentous fungi *P. chrysogenum* and *A. niger*, which were most represented in archives and libraries [162]. These studies indicate that NTP is a promising alternative to other convective methods of inactivating fungal contamination of historical objects.

### 3.4. Plasma in Biotechnology

In the previous sections, we presented many studies focused mainly on the inhibition and inactivation of fungi in various industries such as medicine, agriculture, and food control. However, not all fungal genera are undesirable for humans. Many fungal species produce interesting substances (antibiotics, pigments, and enzymes). In recent years, studies have been emerging investigating the positive effect of NTP on beneficial fungi. Improving the beneficial aspects of fungi using plasma occurs in two ways, through mutagenic or non-mutagenic changes. Studies using plasma for mutagenesis of fungal cells are summarized in Table 3.

Most studies [175,176,177,178,179] used the ARTP plasma mutation system, formed by a radiofrequency atmospheric-pressure glow discharge plasma jet, to mutagenize fungal cells [180]. The *Saccharomyces cerevisiae* mutant prepared by the ARTP mutation system produced approximately 57% more glutathione, and an improvement in glutathione synthetase activity was also observed [181]. After chemical–physical mutagenesis, *Rhodotorula mucilaginosa* K4, with a 67% greater concentration of carotenoids than *Rhodotorula mucilaginosa* KC8, was obtained [178]. The mutated strain JNDY-13, which was obtained with *T. reesei* RUT-C30 as the parental strain, had an increased production of cellulases, which may be related to a mutation in the galactokinase gene. Upregulation of cellulase and hemicellulase genes was also noted in this mutant [174]. In the *C. tropicalis* mutant, in addition to an increase in xylitol production, an increase in xylose reductase gene expression and activity was observed [170]. Feng et al. [171] applied to *A. oryzae* KA-11 a combined mutagenesis program that included microwave mutagenesis, UV irradiation, heat-LiCl, and ARTP. Kojic acid production was increased by 47.0%, 87.1%, 126.2%, and 292.3% compared to the starting strain KA-11 after each stage of mutagenesis. From the obtained results, it is clear that the best results were obtained with ARTP mutagenesis.

Several studies focused on improving spore germination and protein secretion in a non-mutagenic way. A study by Farasat et al. [182] evaluated the effect of NTP on the production of recombinant phytase in the yeast *Pichia pastoris*, as well as the structure and function of the phytase enzyme. The yeast produced higher amounts of recombinant phytase after direct or indirect exposure to plasma. Plasma treatment of a commercial phytase solution with NTP caused up to a 125% increase in enzyme activity. It was also shown that this protein maintained its secondary structure after plasma treatment, while the tertiary structure was slightly changed. Veerana et al. treated *A. oryzae* cells with two plasma discharges, specifically a micro dielectric barrier discharge (MDBD) in nitrogen [183] and a plasma jet in the air [184]. Using MDBD plasma, they achieved a significant increase in the percentage of spore germination after 2 and 5 min of treatment. They also observed a 7.4–9.3% increase in α-amylase activity 24 and 48 h after plasma treatment [183]. After treatment with a plasma jet, they noted an approximately 10% increase in spore germination after 5 and 10 min of treatment and a significant increase in α-amylase activity 24–96 h after plasma treatment [184].

## 4. Summary and Prospects

**Figure 2:**
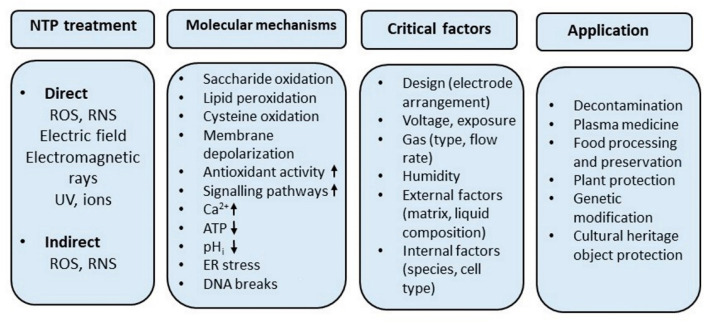
NTP and fungi: treatment setups, applications, and fungal response.

Most studies explored NTP’s application in fungal decontamination, plasma medicine, seed protection, fungal breeding, food processing, preservation, and cultural heritage protection. Despite many advantages that could be exploited, we seem to have reached a point where we must carefully evaluate the positives and negatives when applying this technology to treat fungi. The research concerning fungi and plasma is even more complex because it involves various plasma source configurations, dose determination, working gas compositions, biological and nonbiological matrixes, or liquids (Figure 2).

When working with filamentous fungi, we face many challenges that stem from fungal diversity, the ability to form complex structures, and the formation of hundreds of types of cells that respond to plasma treatment differently. On the one hand, NTP could help combat the emergence of novel pathogens and antifungal-resistant strains by reducing antifungal agents. Nevertheless, on the other hand, the generation and potential spread of genetically modified strains should be of concern when the large-scale employment of NTP is planned. In the future, we have to address not only technical challenges. We must also fill those gaps in understanding the molecular mechanisms involved in fungal interactions with reactive species present in plasma.

## Figures and Tables

**Table 1:** Studies about fungal inactivation, growth inhibition, and biofilm formation.

NTP Type	Process Gas	Time of Treatment	Fungus/Yeast	Effect	Ref.
RF plasma jet	A mixture of argon and oxygen	1–10 min	*Aspergillus flavus*	100% inhibition of growth after 10 min treatment	[69]
Plasma jet	Argon	0–10 min	*Candida parapsilosis* *Magnusiomyces magnusii* *Saccharomyces cerevisiae* *Schizosaccharomyces pombe*	More than 90% inactivation of yeast cells after 10 min	[70]
Plasma microjet	A mixture of helium and oxygen	0–5 min	*Saccharomyces cerevisiae*	The survival ratio of cells in water was significantly decreased from 40.2% to 1.5% after 5 min	[71]
PAW with the plasma jet	Air	1–6 min water activation by plasma	*Aspergillus brasiliensis*	The spore viability dropped to 15% after 30 min in the PAW with a plasma activation time of 3 min	[72]
Linear micro discharge plasma jet	Helium	1 min	*Candida albicans*	Changes in both the genotype and phenotype	[73]
DBSD plasma	Air	0–480 s	*Aspergillus flavus*	A 5 log reduction of spore viability after 480 s under both the low and high power	[56]
Surface micro-discharge plasma	Helium	0–10 min	*Saccharomyces cerevisiae*	The reduction in CFU was about 3.4 log after plasma treatment for 10 min	[74]
DBD plasma	Argon	0–60 min	*Aureobasidium pullulans*	The non-melanized cells were efficiently inactivated, and more than 60% of melanized cells were still alive after the 60 min exposure	[75]
PAW with the CD plasma jet	Air or 99,99% oxygen	0–30 min	*Colletotrichum gloeosporioides*	96% inactivation after 30 min incubation in air-PAW; 55% inactivation after 30 min incubation in oxygen-PAW	[76]
Electric shock-free plasma jet	Air	0–6 min	*Cordyceps pruinosa*	~100% inactivation of spore viability after 6 min	[77]
CD plasma, DBD plasma	Air	0–30 min	*Alternaria* sp. *Aspergillus oryzae* *Byssochlamys nivea* *Cladosporium sphaerospermum*	Spore inhibition after 10–40 min	[78]
Plasma jet	Helium	0–180 s	*Candida albicans*	20–30 mm^2^ inhibition zone area after 180 s	[79]
Plasma jet	Argon	0–180 s	*Neurospora crassa*	Only ~5% spore viability after 3 min in water	[80]

CD, corona discharge; DBD, dielectric barrier discharge; DBSD, dielectric barrier surface discharge; PAW, plasma-activated water; RF, radiofrequency.

**Table 2:** The application of plasma in food and agriculture.

NTP Type	Process Gas	Time of Treatment	Treated Sample	Fungus/Yeast	Effect	Ref.
DBD plasma	Air	0–9 min	Mango	*Colletotrichum asianum*	The disease incidence and lesion diameter of mango treated for 9 min were decreased by 48.00% and 62.95%, respectively	[123]
Plasma jet	Argon, oxygen, nitrogen	0–6 min	Mung bean	Natural fungal contamination	Reduction in natural fungal contamination ranging from 0.54 to 7.09 log at 96 h incubation	[124]
Gliding arc plasma	Nitrogen	300–600 s	Tomato juice	*Candida albicans* *Saccharomyces cerevisiae*	600 s treatment—reduction in fungal viability below the limit of quantification; extension of shelf life to 10 days	[113]
PAW	Air	30–120 s	Kimchi cabbage	Natural fungal contamination	PAW treated with plasma for 120s caused a 1.8 log CFU/g reduction in fungal contamination	[125]
RF cold plasma	Oxygen	0–15 min	Saffron	*Aspergillus* sp. *Penicillium* sp. *Rhizopus* sp.	Complete reduction in contamination after 15 min of treatment	[126]
Microwave plasma	Helium	40 min	Onion powder	*Aspergillus brasiliensis*	1.6 log spores/cm^2^ reduction	[127]
Flexible thin-layer plasma	Air	10 min	Beef jerky packaged	*Aspergillus flavus*	2–3 log CFU/g reduction in spore viability	[106]
DBD plasma	Air	0–5 min	Citrus	*Penicillium venetum* *Aspergillus brasiliensis*	Significantly decreased to ~1.50 log CFU/mL at 2 min; significantly decreased viable count to ~1.62 log CFU/mL at 5 min	[128]
DBSD plasma	Air	0–20 min	Blueberry	*Botrytis cinerea*	15 and 20 min plasma treatment completely inhibited the mycelial growth	[115]
Microwave plasma	Air	15–60 s	Allspice berry Black pepper Juniper berry	*Aspergillus niger*	Partial inactivation after 15 s treatment	[129]
Gliding arc plasma	Humid argon	0–7 min	Mango	*Colletotrichum gloeosporioides*	Significantly lower mycelium growth rate constant, the maximum reduction in spores was 1 log spore/mL after 7 min of NTP treatment with 5 L/min gas flux	[130]
PAW with the plasma jet	Air	0–30 min	Mung bean sprout	Natural yeast contamination	~2.8 log CFU/g yeasts reduction after 30 min	[118]
Plasma jet	Air	0–90 s	Paprika	*Fusarium oxysporum*	Complete inhibition of mycelial growth and spore germination after 90 s of treatment but only 50% inhibition of fungal growth on the paprika surface	[117]
AP plasma jet; LP RF plasma	Air, nitrogen, Oxygen	0–30 min	Hazelnut	*Aspergillus flavus* *Aspergillus parasiticus*	Spore reductions of 4.7 and 5.6 log CFU/g after 30 min of LP air plasma treatment; spore reductions of 5.4 and 5.5 log CFU/g after 1.7 min of AP air plasma treatment; deformation of spores and loss of spore integrity after plasma treatments	[131]
DBD plasma	Air	0–10 min	Blueberry	natural fungal contamination	The number of fungi decreased by 25.8%; the blueberry decay rates were reduced by 5.2% in the plasma treatment of 10 min after 20 days of storage	[132]
CD plasma jet	Air	0–120 s	Kumquat	natural yeasts contamination	0.77–1.57 log CFU/g reduction after 120 s treatment	[116]
Fluidized bed plasma	Air, nitrogen	0–5 min	Hazelnuts	*Aspergillus flavus* *Aspergillus parasiticus*	~4 log fungicidal effects after 5 min; the air plasma was more effective than nitrogen plasma	[133]
Surface barrier discharge	Air	0–8 min	Corn kernels	-	Complete degradation of aflatoxin B1 after 6 min of treatment	[134]
DBD plasma	Air	0–180 s	Pistachio nuts, glass slides	*Aspergillus flavus*	Decrease in spore population by 4 log after 180 s of the treatment; maximum reduction in AFB1 was observed after 180 s of the treatment, which was 64.63% for glass slides and 52.42% for pistachio nuts	[135]
CD plasma jet	Air	0–30 min	Rice, Wheat	-	Initial AFB1 concentration on slides was decreased maximally by 95% in 30 min; in rice and wheat, the average levels of AFB1 degradation ranged between 45 and 56% following 30 min treatment	[136]
AP plasma jet; LP RF plasma	Air	0–30 min	Hazelnuts	-	Both plasmas reduced 72–73% of AFB1 spiked on hazelnuts after plasma treatment	[137]
RF plasma	air with H_2_O_2_ (35%)	0–10 min	Cannabis inflorescences	*Botrytis cinerea*	5 log reduction in viable fungal spores after 10 min	[138]
DBD plasma	Argon or a mixture of 80% argon and 20% oxygen	10 min	Ginseng seeds	natural fungal contamination from the surface of seeds	~73% (Ar) and 60% (Ar/O_2_) inactivation of fungal spores	[139]
DBSD plasma	Air	0–60 s	Scot pine seeds	*Fusarium oxysporum*	100% disinfection efficiency of seeds after 30 s treatment	[140]
DCSBD plasma	Air	0–10 min	Lentil seeds	*Aspergillus niger* *Penicillium decumbens*	Maximum logarithmic reduction of 1.6 log CFU/g for *A. niger* and 3.1 log CFU/g for *P. decumbens* after 10 min	[141]
DBD plasma	Nitrogen, Oxygen	1–3 min	Soybean seeds	*Diaporthe/Phomopsis complex*	Reduction in infection by about 49–81%	[142]
DBD plasma	Air	5, 20 min	Barley and wheat seeds	*Penicillium verrucosum*	Maximal reduction of 2.1 log CFU/g for barley and 2.5 log CFU/g for wheat	[67]
DCSBD plasma	Air	0–50 s	Cucumber and pepper seeds	*Cladosporium cucumerinum* *Didymella bryoniae* *Didymella licopersici*	Total reduction in *C. cucumerinum* and 60–80% reduction in *D. bryoniae* on cucumber seeds after 20 s; 50–80% reduction in *D. licopersici* on pepper seeds	[143]
DCSBD plasma	Air	0–300 s	Maize	*Alternaria alternata* *Aspergillus flavus* *Fusarium culmorum*	Reduction of 3.79 log CFU/g in *F. culmorum* after 60 s plasma treatment, 4.21 log CFU/g in *A. flavus*, and 3.22 log CFU/g in *A. alternata* after a 300 s plasma treatment	[66]
DBSD plasma	Air	0–300 s	Sweet basil seeds	natural fungal contamination	~30% reduction of natural fungal contamination after 300 s	[144]
Plasma jet	Humid air	10 min	Rice seeds	*Fusarium fujikuroi*	Bakanae disease severity index and the percentage of plants with symptoms were reduced to 18.1% and 7.8% of nonirradiated control, respectively, after 10 min treatment of seeds in water	[145]
RF plane-type plasma	Air	0–30 min	Groundnuts	*Aspergillus flavus* *Aspergillus parasiticus*	High percentage of inactivation, 99.9% and 99.5% of *A. parasiticus* and *A. flavus*, respectively	[146]
CD plasma jet	Air	0–3 min	Broccoli seeds	natural fungal contamination	1.5 log CFU/g reduction in natural fungal contamination after 3 min	[147]

AP, atmospheric pressure; CD, corona discharge; DBD, dielectric barrier discharge; DBSD, dielectric barrier surface discharge; DCSBD, diffuse coplanar surface barrier discharge; LP, low pressure; PAW, plasma-activated water; RF, radiofrequency.

**Table 3:** Studies using NTPs for mutagenesis of fungi.

NTP Type	Process Gas	Time of Treatment	Fungus/Yeast	Mutant	Ref.
ARTP	Helium	0–180 s	*Fusidium coccineum*	~60%increase in fusidic acid production	[163]
ARTP	Helium	0–350 s	*Aspergillus nidulans*	1.3× higher production of echinocandin B	[164]
ARTP	Helium	-	*Sanghuangporous sanghuang*	1.2–1.5 fold increase in polysaccharides production	[165]
ARTP + etylmethanesulfonate	Helium	0–550 s	*Penicillium oxalicum*	Enhanced raw starch-degrading enzyme production	[166]
ARTP	Helium	30–240 s	*Aspergillus oryzae*	54.7% increase in acid protease activity, 17.3% increase in neutral protease activity, 8.5% increase in total protease activity, 8.1% decrease in alkaline protease activity	[167]
ARTP	Helium	0–360 s	*Starmerella bombicola*	30% increase in lactonic, acidic, or total sophorolipid production	[168]
ARTP	Helium	0–200 s	*Candida parapsilosis*	~60% increase in D-arabitol production	[169]
ARTP	Helium	0–150 s	*Candida tropicalis*	22% increase in xylitol production	[170]
ARTP	Helium	100–200 s	*Aspergillus oryzae*	~292% increase in kojic acid production	[171]
ARTP	Helium	30 s	*Hericium erinaceum*	22% increase in yield of fruiting body, 16% increase in polysaccharide production	[172]
DBD plasma	Argon helium	3–5 min	*Ganoderma lingzhi*	25.6% increase in polysaccharides production	[173]
ARTP	Helium		*Trichoderma reesei*	Increase in cellulase production	[174]

ARTP, atmospheric and room temperature plasma; DBD, dielectric barrier discharge.

## Data Availability

Not applicable.
